# Impaired TrkB Signaling Underlies Reduced BDNF-Mediated Trophic Support of Striatal Neurons in the R6/2 Mouse Model of Huntington’s Disease

**DOI:** 10.3389/fncel.2016.00037

**Published:** 2016-03-09

**Authors:** Khanh Q. Nguyen, Vladimir V. Rymar, Abbas F. Sadikot

**Affiliations:** Cone Laboratory, Department of Neurology and Neurosurgery, Montreal Neurological Institute, McGill UniversityMontreal, QC, Canada

**Keywords:** brain-derived neurotrophic factor (BDNF), DARPP-32, Huntington disease, R6/2, striatum, TrkB

## Abstract

The principal projection neurons of the striatum are critically dependent on an afferent supply of brain derived neurotrophic factor (BDNF) for neurotrophic support. These neurons express TrkB, the cognate receptor for BDNF, which activates signaling pathways associated with neuronal survival and phenotypic maintenance. Impairment of the BDNF-TrkB pathway is suspected to underlie the early dysfunction and prominent degeneration of striatal neurons in Huntington disease (HD). Some studies in HD models indicate that BDNF supply is reduced, while others suggest that TrkB signaling is impaired earlier in disease progression. It remains important to determine whether a primary defect in TrkB signaling underlies reduced neurotrophic support and the early vulnerability of striatal neurons in HD. Using the transgenic R6/2 mouse model of HD we found that prior to striatal degeneration there are early deficits in striatal protein levels of activated phospho-TrkB and the downstream-regulated protein DARPP-32. In contrast, total-TrkB and BDNF protein levels remained normal. Primary neurons cultured from R6/2 striatum exhibited reduced survival in response to exogenous BDNF applications. Moreover, BDNF activation of phospho-TrkB and downstream signal transduction was attenuated in R6/2 striatal cultures. These results suggest that neurotrophic support of striatal neurons is attenuated early in disease progression due to defects in TrkB signal transduction in the R6/2 model of HD.

## Introduction

Huntington’s disease (HD) is a progressive autosomal dominant disease characterized by early hyperkinetic movements and behavioral changes (Roze et al., 2011). HD patients carry an expanded CAG-repeat in exon 1 of the IT15/HTT gene that yields an elongated polyglutamine stretch in the N-terminal domain of the huntingtin (htt) protein (Bates, 2005). A hallmark of HD is the early degeneration of medium spiny neurons (MSNs) in the striatum, which constitute >95% of the neuronal population in the striatum. MSNs are the principal projection neurons of the striatum, which is the initial relay nucleus of the basal ganglia network that processes cortical and thalamic glutamatergic and midbrain dopaminergic signals that regulate locomotor and motivational behavior (Tepper et al., 2007). In HD patients and animal models *mutant huntingtin* (*mhtt*) is expressed by a wide variety of neuronal populations with no clear preference for the vulnerable MSNs population (Trottier et al., 1995; Bhide et al., 1996). Therefore the early degeneration of MSNs in HD may be related to striatum-specific cellular processes that are sensitive to the presence of *mhtt*. Evidence in cellular and animal models of HD and in HD (Raymond et al., 2011; Francelle et al., 2014) indicate *mhtt* expression is associated with forebrain abnormalities in glutamatergic and dopaminergic transmission (Bibb et al., 2000; Cepeda et al., 2014), dysregulation of gene transcription (Neueder and Bates, 2014), altered striatal enriched proteins (e.g., PDE10A, STEP, Rhes; Subramaniam et al., 2009; Saavedra et al., 2011; Leuti et al., 2013; Mealer et al., 2014; Fusco and Giampà, 2015) and impaired neurotrophic support (e.g., BDNF-TrkB pathway; Zuccato et al., 2001; Wild and Tabrizi, 2014). Importantly, since the survival and maintenance of MSNs are especially dependent on the BDNF-TrkB signaling pathway (Ivkovic and Ehrlich, 1999; Baydyuk et al., 2011), reduced neurotrophic support may underlie the early vulnerability of MSNs to degeneration in HD.

Striatal MSNs express TrkB, the cognate receptor for brain derived neurotrophic factor (BDNF), but do not produce BDNF. They therefore rely on a supply from cortical, dopaminergic and thalamic afferents for neurotrophic maintenance of survival and phenotypic function (Altar et al., 1997; Sadikot et al., 2005). Low BDNF protein levels are reported in postmortem striatal tissue from HD patients at symptomatic disease stages (Ferrer et al., 2000; Zuccato et al., 2008), however, it is not clear whether this is associated with early neurtrophic deficiencies for MSNs or, rather, a consequence of late stage cortical and striatal neuron atrophy. Cellular models show that BDNF transcription and transport can be impaired by overexpression or knock-in of *mhtt* (Zuccato et al., 2001; Gauthier et al., 2004). Accordingly, *in vivo* deficits of striatal BDNF levels are shown to correlate with reduced cortical BDNF mRNA expression in some studies of Q175 knock-in and YAC72 transgenic HD mouse models (Zuccato et al., 2001; Ma et al., 2015). In contrast, evidence of normal striatal BDNF levels at early and intermediate disease stages in these (Seo et al., 2008; Plotkin et al., 2014) and other HD mouse models (Pang et al., 2006; Potenza et al., 2007; Traficante et al., 2007; Seo et al., 2008; Cepeda et al., 2010; Bobrowska et al., 2011) argues that defects further downstream may underlie early deficiencies in neurotrophic support for MSNs in HD.

BDNF neurotrophic support of MSNs is mediated by full-length (fl) TrkB, a member of the neurotrophic tyrosine receptor kinase family, that activates cytosolic signaling cascades that promote survival and maintain neurochemical and morphological properties. Low striatal fl-TrkB protein levels are reported in postmortem tissue from HD patients at late disease stages (Ginés et al., 2006; Brito et al., 2013). Studies on HD mouse models have demonstrated varying degrees of striatal TrkB deficits. Normal levels are reported at early disease stages in knock-in Q175 (Smith et al., 2014; Ma et al., 2015) and transgenic R6/1, R6/2, BACHD and D9 mice (Brown et al., 2008; Gharami et al., 2008; Martire et al., 2010; Brito et al., 2013; Plotkin et al., 2014), while deficits are reported at early stages in knock-in HdhQ111 mice and later stages in R6/1 mice (Brito et al., 2013). As an important caveat, observations at later stages may be confounded by loss of volume or number of striatal neurons as disease progresses (Stack et al., 2005; Samadi et al., 2013). On the other hand, normal TrkB levels observed at early stages may not necessarily reflect normal receptor function. For example, defects in transport and scaffolding proteins associated with TrkB signal transduction down the Erk1/2 pathway are demonstrated in *mhtt* knock-in STHdhQ111 cells and HdhQ111 striatal cultures independent of changes in TrkB expression (Ginés et al., 2010; Liot et al., 2013). The variability of reports on TrkB expression and signaling in HD models may be attributed in part to suboptimal tissue preservation and also fundamental differences in cellular and mouse models. Examining TrkB receptor expression in a well-characterized transgenic *mhtt* model and further characterizing BDNF functional activation of TrkB signaling in primary striatal cultures derived from the same model may better define which defects occur in the BDNF-TrkB pathway and how this affects neurotrophic support of MSNs at early disease stages of HD.

We determined whether a primary defect in fl-TrkB expression or signaling underlies reduced trophic support of striatal neurons at an early stage in the R6/2 mouse model of HD. The R6/2 mouse carries a 5′ end of *exon* 1 of mutant human HTT transgene with expanded CAG repeats that produces an N-terminal *mhtt* fragment protein. The R6/2 mouse exhibits HD-like locomotor disorder and striatal pathology that is accelerated relative to other genetic mouse models of HD (Mangiarini et al., 1996; Menalled et al., 2009; Samadi et al., 2013). Our assays on presymptomatic R6/2 mice indicated that striatal morphology and protein levels of BDNF and fl-TrkB are normal but levels of activated phospho-TrkB are reduced compared to WT mice. Possible defective BDNF-TrkB signaling was therefore further investigated *in vitro* using primary striatal neuron cultures derived from R6/2 mice. We found that the normal trophic effects of BDNF on striatal neurons were attenuated in R6/2 cultures. Moreover, this was associated with impaired BDNF-mediated activation of phospho-TrkB despite normal expression of fl-TrkB in R6/2 cultures. Finally, downstream activation of Erk1/2 was also impaired in R6/2 cultures. Together these findings suggest that an early impairment of TrkB activation and downstream signaling underlies reduced trophic support of striatal neurons in HD.

## Materials and Methods

### Animals

Wildtype (WT) B6CBA males were bred with female mice that had received ovarian transplants from R6/2 females, which carry exon-1 of a human mutant HTT transgene with 120–130 CAG repeats (The Jackson Laboratory, MA, USA). Animal procedures were in accordance with the Canadian Council on Animal Care guidelines for ethical use and welfare of animals in research, as administered by the McGill University Animal Care Committee. R6/2-ovarian transplanted females were mated with WT males to obtain littermate pups of both genotypes. Tail-tip samples from pups were used for PCR genotyping, and CAG repeat size (125 ± 5 repeats) was verified. Littermates of both sexes were used at either at postnatal day (P) 28–35 for striatal morphology and protein assays, or at P1 for primary striatal neuron culture assays.

### Tissue Preparation for Striatal Stereology and Protein Assays

Littermate adolescent pups (P28–35) were sacrificed by decapitation for protein assays or by deep anesthesia with phenobarbital and transcardial perfusion (0.9% heparinized saline followed by PFA fixation- 4% paraformaldehyde in 0.1 M phosphate buffer, pH 7.4) for stereology. Brains for stereological analysis were cryoprotected and subsequently sectioned at 40 μm in the coronal plane then processed for Nissl-staining (as previously described in detail, Samadi et al., 2013). The neostriatum was delineated according to defined boundaries (Sadikot and Sasseville, 1997) using a stereotaxic atlas of the mouse brain (Franklin and Paxinos, 2008). The optical fractionator or nucleator were used as stereology probes to obtain unbiased estimates of the total number of neurons or cell size, respectively, for individual animals and the results were averaged according to genotype (as detailed in our previous work, Luk et al., 2003; Rymar et al., 2004). For protein analysis, unperfused brains were dissected into −80°C isopentane. Atlas defined (Franklin and Paxinos, 2008) dorsolateral striatum tissue samples were obtained using a 1 × 1.5 mm cylindrical micro-punch (Stoelting, IL, USA). Striatal punches were lysed in 60 μL of RIPA buffer containing protease and phosphatase inhibitors and assessed by western blot (see below).

### Primary Striatal Cultures

Previous studies characterizing the viability and phenotype of striatal cultures indicate higher neuronal survival in B27- compared to N2-supplemented neurobasal media (NBS; Brewer, 1995; Ivkovic and Ehrlich, 1999). N2 lacks vitamin A and free radical scavenging enzymes: catalase, glutathione and superoxide dismutase that are known trophic components of B27 (Ivkovic and Ehrlich, 1999). Therefore our striatal culture assays were done in N2-NBS, which minimizes confounding trophic effects of B27. Postnatal day 1 (P1) striatal tissue from individual pups was processed into parallel sister culture wells on poly-lysine coated 96- or 6-well plates (BD Inc., ON, Canada). Using microscissors and a dissecting microscope, striatal tissue was dissected into ice-cold B27-Hibernate-A media (Invitrogen, ON, Canada), digested with Tryp-LE and finally dissociated in B27-NBS media (Invitrogen). Dissociated cells were plated at low density (400 cells/mm^2^; 96-well poly-D-lysine coated plates) or high density (obtain sufficient protein concentrations for western blot assays, 1000 cells/mm^2^; 6-well poly-D-lysine coated plates) and allowed to attach for 24 h in B27-NBS. After 1 day *in vitro* (DIV) the low density cultures were switched into N2-NBS with vehicle (PBS) or BDNF (2, 10, 50 or 100 ng/mL) and grown to 7 DIV, and finally used for immunofluorescence cell counting assays. High density cultures were switched to N2-NBS and grown to 3 DIV and used for protein quantification assays.

### Immunofluorescence Staining

BDNF effects on neuronal viability and phenotype in WT and R6/2 cultures were assessed by immunofluorescence staining for neurochemical markers of various cellular subpopulations. On a single 96 well plate, sister wells (24 wells) from individual pups (at least one WT and one R6/2 pup per plate) were divided into four dose groups: 0, 2, 10 and 50 ng/mL BDNF in N2-NBS media. Media containing the different BDNF doses was replenished every 3 days. After 7 DIV, wells were fixed with 4% paraformaldehyde buffer (4% paraformaldehyde in 0.1 M phosphate buffer, pH 7.4), then blocked in 4% BSA-PBS. The total cell population was labeled using the nuclear stain DAPI (1:1000; Roche Inc, QC, Canada). The neuronal population was co-labeled for β-III-tubulin (Tuj1 antibody, 1:3000; Covance, NJ, USA). Alexa-564 goat anti-rabbit antibody was used for secondary fluorescent conjugation (1:1000; Invitrogen). The apoptotic cell population was labeled using a TUNEL staining kit (Roche Inc.). Other sets of cultures were similarly plated and immumostained for makers of other cellular subpopulations: striatal neuron markers- mouse anti-MAP2 (1:5000; Sigma Aldrich, ON, Canada) and rabbit anti-DARPP-32 (1:500; Millipore, MA, USA); mitotic progenitors- rabbit Ki67 (1:1000; Millipore). Fluorescence images were acquired for each culture well at 16 predefined sites (10× objective, XCD microscope; Molecular Devices, PA, USA). MetaXpress software (Molecular Devices) was used to delineate and count various immuno-labeled cell populations. The densities and proportions (relative to total DAPI+ cell density) of each labeled population was calculated per well, then averaged according to treatment group and reported with standard errors.

### Western Blotting

Protein lysates were made from striatal tissue punches (see above) or primary striatal cultures from individual WT and R6/2 animals. Primary striatal cultures (see above) were grown in N2-NBS for 48 h and then acutely exposed to control (0 ng/ml; 20 min) or BDNF (100 ng/ml; 20 min) supplemented media. Cultures were washed with PBS and total-protein lysates were obtained in RIPA buffer containing protease and phosphatase inhibitors and assessed by standard western blot methods (Luk and Sadikot, 2004). Equivalent volumes of lysate from WT and R6/2 tissue punches (~20 mg of total protein) or from cultures (~2 mg of total protein) were ran in parallel on 4–15% gradient gels for SDS-PAGE. Recombinant BDNF protein (PeproTech, NJ, USA) was also run in parallel as a marker for the migration of endogenous BDNF at 14 kD (Fawcett et al., 1997). Nitrosecellulose blots were blocked with 2.5% BSA-TBST and then probed with antibodies for total TrkB (1:1000; Cell Signaling Technologies, MA, USA, #4603), Erk1/2 (1:1500; CST, #9102), DARPP-32 (1:2000; Millipore, #ab1656) and β-III-tubulin (1:10,000; Covance, #PRB435). Replicate blots were generated in parallel and probed for activated signaling proteins phospho-TrkB (Tyr704; 1:500; Santa Cruz, CA, USA, #sc135645), phospho-Erk1/2 (Thr202/204; 1:2000; CST, #9106) and β-III-tubulin. Immunoblots were labeled with secondary HRP-conjugated antibodies (goat anti-rabbit or-mouse; 1:10,000; Millipore) and developed by ECL (Pierce, IL, USA) and exposure on X-ray film (VWR, ON, Canada). Films were scanned on a flatbed scanner with backlighting. Image-J (NIH software, version 1.47) was used for optical density (OD) quantification of each protein band. Relative protein levels were derived by normalizing the OD of the specific band to that of the β-III-tubulin (Tuj1) band in the same lane, thereby controlling for any effect of unequal total neuronal protein concentrations between lanes. Normalization of phospho-proteins to their total unphosphorylated forms yielded similar results as normalization to β-III-tubulin (data not shown).

### Statistical Analysis

Stereology and protein assays were done on brains of individual WT (*n* > 3) and R6/2 (*n* > 3) mice and the data were averaged according to genotype and reported as mean ± standard error (SE). Mean data were compared for significant differences (*p* < 0.05) using the Student’s *t*-test. Immunofluorescence assays of BDNF dose-effects were done on parallel sister cultures (>16 culture wells/individual animal) derived from individual WT (*n* > 3) and R6/2 (*n* > 3) mice. Data were grouped according to genotype and BDNF dose (control, 2, 10, 50 ng/mL) and reported as mean ± SE. ANOVA was used to determine the main effects of genotype and drug, and posthoc Fisher-LSD comparisons were done to determine significant differences between genotypes at each dose. Protein assays of the effect of BDNF on parallel sister cultures (2 culture wells/animal/2 drug doses) that were derived from individual WT (*n* = 6) and R6/2 (*n* = 5) mice. Data were grouped according to genotype and BDNF dose (control or 100 ng/mL) and reported as mean ± SE. Mean data were compared between genotype groups at each drug dose for significant differences (*p* < 0.05) using the Student’s *t-test*.

## Results

### Normal Morphology of Striatum in Young R6/2 Mice

**Figure 1:**
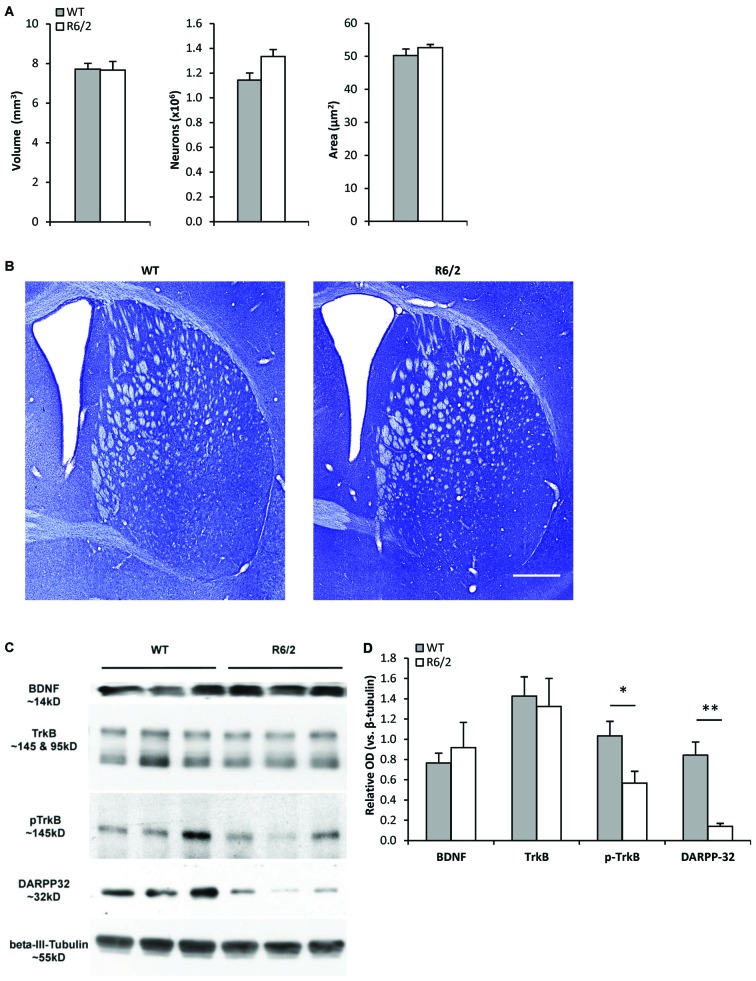
**Young R6/2 mice exhibit normal striatal morphology but reduced phospho-TrkB protein levels. (A)** Stereological estimates of total volume of the neostriatum, number of striatal neurons, and cross-sectional area of striatal neurons in WT (*n* = 3) and R6/2 (*n* = 3) mice. **(B)** Photomicrographs of Nissl-stained coronal sections of striatum from a WT and R6/2 mouse. Scale bar = 1500 μm. **(C)** Sample immunoblots of proteins in striatal tissue lysates from different WT and R6/2 mice. **(D)** BDNF and full-length (fl) TrkB levels were similar in WT (*n* = 7) and R6/2 (*n* = 6) striatal tissue. Significantly lower levels of phospho-TrkB and DARPP-32 are noted in R6/2 compared to WT striatal tissue. Relative protein levels were determined by normalization to the neuronal specific cytoskeleton protein β-III-tubulin. Unpaired Student’s *t*-test: *p < 0.05, **p < 0.001.

Unbiased stereology (Figures 1A,B) was used to determine whether R6/2 mice exhibited striatal morphological changes at a young age (P28–35) associated with a presymptomatic disease stage (Samadi et al., 2013). The estimated total number of neurons in the striatum was similar in WT and R6/2 mice (1,144,207 ± 55,809 vs. 1,333,955 ± 55,522 cells, respectively; *p* > 0.05). The cross-sectional area of striatal neurons was also similar in WT and R6/2 mice (50.3 ± 2.0 vs. 52.6 ± 0.9 μm^2^, respectively; *p* > 0.05). Moreover, the total striatal volume was similar in WT and R6/2 mice (7.72 ± 0.28 vs. 7.67 ± 0.43 mm^3^, respectively; *p* > 0.05).

### Reduced Phospho-TrkB Levels in the Striatum of Young R6/2 Mice

Western blot analysis of striatal tissue punches from young (P28–35) R6/2 mice were assessed for alterations in components of the BDNF-TrkB pathway. Striatal BDNF and fl-TrkB levels were found to be similar in R6/2 and WT mice (Figures 1C,D). Levels of truncated-TrkB (95 kDa; Figure 1C) were also similar in R6/2 and WT mice (data not shown). However, levels of activated phospho-TrkB were lower in R6/2 compared to WT mice (Figures 1C,D). Since activation of phospho-TrkB signaling regulates expression of the dopamine and cAMP regulated neuronal phosphoprotein 32 kDa (DARPP-32; an MSN-specific protein; Ivkovic and Ehrlich, 1999), we also found that striatal DARPP-32 levels were low in young R6/2 mice (Figures 1C,D).

### Normal Basal Viability and Neuronal Phenotype of R6/2 Striatal Cultures

**Figure 2:**
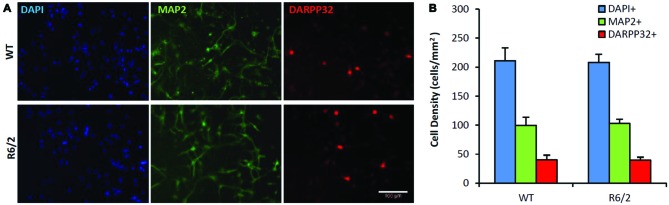
**Similar neuronal populations in WT and R6/2 striatal cultures in basal media conditions. (A)** Co-immunofluorescence images of DAPI+ (blue), MAP2+ (green) and DARPP-32+ (red) stained cells in WT (top panels) and R6/2 (bottom panels) cultures after 7 day *in vitro* (DIV) in unsupplemented media. Scale bar = 100 μm. **(B)** Average density of cells expressing neuronal (MAP2+) and striatal (DARPP-32+) phenotype in WT (*n* = 19) and R6/2 (*n* = 19) cultures.

Sister cultures (*n* = 19/genotype group) derived from the striatum of individual WT (*n* = 4) and R6/2 (*n* = 4) littermate mice were first assessed for differences in basal cell survival and neuronal phenotype at 7 DIV in unsupplemented media. Previous studies using similar minimal media conditions observe that striatal cultures exhibit a majority population of neurons and a very small population of non-neuronal cells (Mizuno et al., 1994; Nakao et al., 1994; Ventimiglia et al., 1995). Counts of DAPI+ staining, a DNA label that identifies all cell nuclei in culture, revealed that total cell density was similar in WT and R6/2 cultures (211 ± 22 and 208 ± 14 cells/mm^2^, respectively; Figures 2A,B) after 7 DIV in basal media. Concurrently, MAP2+ staining (microtubule associated protein 2; a neuron-specific cytoskeletal protein) indicated a similar sized population of neuronal cells in WT and R6/2 cultures (100 ± 14 and 103 ± 7 cells/mm^2^, respectively; Figures 2A,B). Furthermore, DARPP-32+ staining indicated a similar sized population of MSN-like cells in WT and R6/2 cultures after 7 DIV in basal media (40 ± 8 and 40 ± 5 cells/mm^2^, respectively; Figures 2A,B). These results are in line with other studies using striatal cultures derived from newborn WT (Ivkovic and Ehrlich, 1999) and R6/2 mice (Petersén et al., 2001), which typically exhibit a small population of DARP-32+ neurons (1–25% of total cells in culture) due in part to striatal tissue dissection at P1 prior to the postnatal developmental period of neurotrophin expression (Fryer et al., 1996; Sadikot et al., 2005) and MSN differentiation (Ivkovic et al., 1997).

Other cellular markers of non-neuronal cells were assessed in separate sets of cultures. GFAP+ staining (glial fibrillary acidic protein) demonstrated a minor population of astrocytes in cultures from both genotypes. The size of the astrocyte population was similar in WT and R6/2 cultures (2.1 ± 0.3 and 1.5 ± 0.4%, respectively; *n* > 15). The mitotic cell population was assessed using Ki67+ staining. The size of the mitotic cell population was also similar in WT and R6/2 cultures (6.7 ± 0.3 and 7.1 ± 0.4%, respectively; *n* > 15).

### BDNF Neurotrophic Effects are Attenuated in R6/2 Striatal Cultures

To determine whether there is a primary defect in TrkB function in HD striatum, sister cultures (*n* > 15/dose group) were derived from the striatum of individual WT (*n* = 3) and R6/2 (*n* = 5) littermate mice and assessed for BDNF dose-effect on neuronal survival. Previous studies have demonstrated that BDNF, via activation of TrkB, mediates trophic effects on striatal neurons in culture. For example, striatal cultures exposed to BDNF (10–100 ng/mL; 7 DIV) exhibit greater neuronal survival and MSN phenotype (e.g., DARPP-32 expression) compared to untreated cultures (Mizuno et al., 1994; Nakao et al., 1995; Ventimiglia et al., 1995).

**Figure 3:**
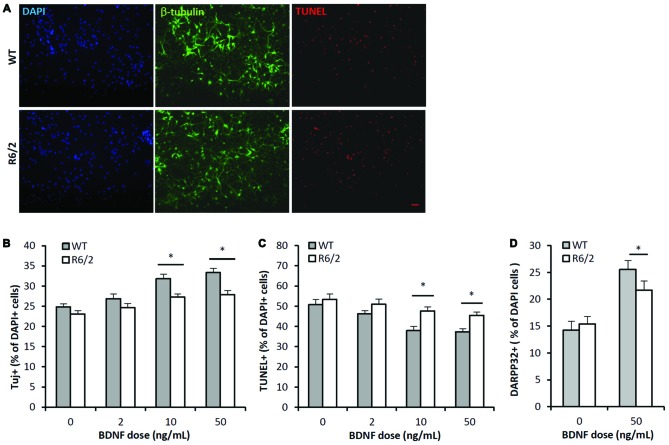
**BDNF neurotrophic effects are attenuated in R6/2 striatal cultures. (A)** Co-immunofluorescence images of DAPI+ (blue), β-III-tubulin+ (green) and TUNEL+ (red) stained cells after 7 DIV in media supplemented with 50 ng/mL BDNF. BDNF promoted the survival of β-III-tubulin+ neurons and attenuated the number of dying TUNEL+ cells in WT cultures (top panels) to a greater extent than in R6/2 cultures (bottom panels). Scale bar = 50 μm. **(B)** BDNF dose-effect on the β-III-tubulin+ neuronal population in WT (*n* = 15–18/dose) and R6/2 (*n* = 25–29/dose) cultures. ANOVA main effects: dose- *F_(3,167)_*, *p* < 0.01; genotype- *F_(1,167)_*, *p* < 0.01; Fisher-LSD comparisons: *p < 0.05. **(C)** BDNF dose-effect on TUNEL+ dying cell population in WT (*n* = 15–18/dose) and R6/2 (*n* = 25–29/dose) cultures. ANOVA main effects: dose- *F_(3,167)_*, *p* < 0.01; genotype- *F_(1,167)_*, *p* < 0.01; Fisher-LSD comparisons: *p < 0.05. **(D)** BDNF effect on DARPP-32+ striatal neuron population in WT (*n* = 19) and R6/2 (*n* = 20) cultures. Unpaired Student’s *t*-test: *p < 0.05.

The BDNF dose-effect (2–50 ng/mL; 7 DIV) on neuronal survival was assessed by quantifying the cellular population that was immunoreactive for β-III-tubulin, a neuron specific cytoskeleton protein. There were significant main effects of both BDNF dose and genotype on the population of neurons in culture (Figures 3A,B; ANOVA: dose- *F_(3,167)_*, *p* < 0.01); genotype- *F_(1,167)_*, *p* < 0.01). Fisher-LSD posthoc pairwise comparisons within the WT genotype group indicated the neuronal populations in the 2, 10 and 50 ng/mL dose groups were larger (*p* < 0.05) than in the untreated WT group. In contrast, comparisons within the R6/2 genotype group indicated that only the 10 and 50 ng/mL dose groups had larger (*p* < 0.05) neuronal populations than the untreated R6/2 group. Fisher-LSD posthoc pairwise comparisons within the control dose (0 ng/mL) group did not detect any differences between WT vs. R6/2 cultures. However, within the 10 and 50 ng/mL dose groups, the neuronal population was larger (*p* < 0.05) in WT compared to R6/2 cultures (e.g., 50 ng/mL: 33.4 ± 0.7 vs. 27.9 ± 1.1%, respectively; Figures 3A,B). Notably, BDNF (50 ng/mL) treatment lead to a ~34% increase in the neuronal population in WT cultures, in contrast to a ~21% increase in R6/2 cultures. Striatal neurons in R6/2 cultures were therefore resistant to BDNF survival effects compared to neurons in WT cultures.

The BDNF dose-effect on apoptosis was assessed by quantifying the population of TUNEL+ stained cells. There were significant main effects of both BDNF dose and genotype on the population of TUNEL+ cells in culture (Figures 3A,C; ANOVA: dose- *F_(3,167)_*, *p* < 0.01; genotype- *F_(1,167)_*, *p* < 0.01). Fisher-LSD posthoc pairwise comparisons within the WT genotype group indicated the TUNEL+ populations in the 10 and 50 ng/mL dose groups was smaller than in the untreated WT group. Also, comparisons within the R6/2 group indicated the TUNEL+ populations in the 10 and 50 ng/mL dose groups were lower than in the untreated R6/2 group. Fisher-LSD posthoc pairwise comparisons between the WT and R6/2 genotypes at the 10 or 50 ng/mL doses indicated that the TUNEL+ populations in WT cultures were lower than in R6/2 cultures (e.g., 50 ng/mL: 37.4 ± 1.5 vs. 45.5 ± 1.6%, respectively; *p* < 0.05). BDNF is therefore less effective in rescuing cells from apoptosis in R6/2 cultures compared to WT cultures.

BDNF-TrkB signaling is well known to promote expression of DARPP-32 (Nakao et al., 1995; Ivkovic et al., 1997), a striatal-enriched protein essential for proper function of striatal MSNs (Bibb et al., 2000). Therefore BDNF-TrkB function was assessed by comparing the DARPP-32+ populations in WT and R6/2 cultures that were exposed to either vehicle or BDNF supplemented media. In vehicle supplemented media the DARPP-32+ population in WT and R6/2 cultures was similar (14.2 ± 1.3 and 15.5 ± 1.0%, respectively; Figure 3D). In BDNF (50 ng/mL) supplemented media the DARPP-32+ population was larger in WT compared to R6/2 cultures (25.5 ± 1.7 and 21.7 ± 1.7%, respectively; *p* < 0.05; Figure 3D). Notably, BDNF treatment lead to a ~76% increase in the DARPP-32+ population in WT cultures in contrast to a ~41% increase in R6/2 cultures. These results indicate that R6/2 striatal cultures are less sensitive to BDNF-stimulated expression of DARPP-32.

### BDNF-TrkB Signal Transduction is Impaired in R6/2 Striatal Cultures

**Figure 4:**
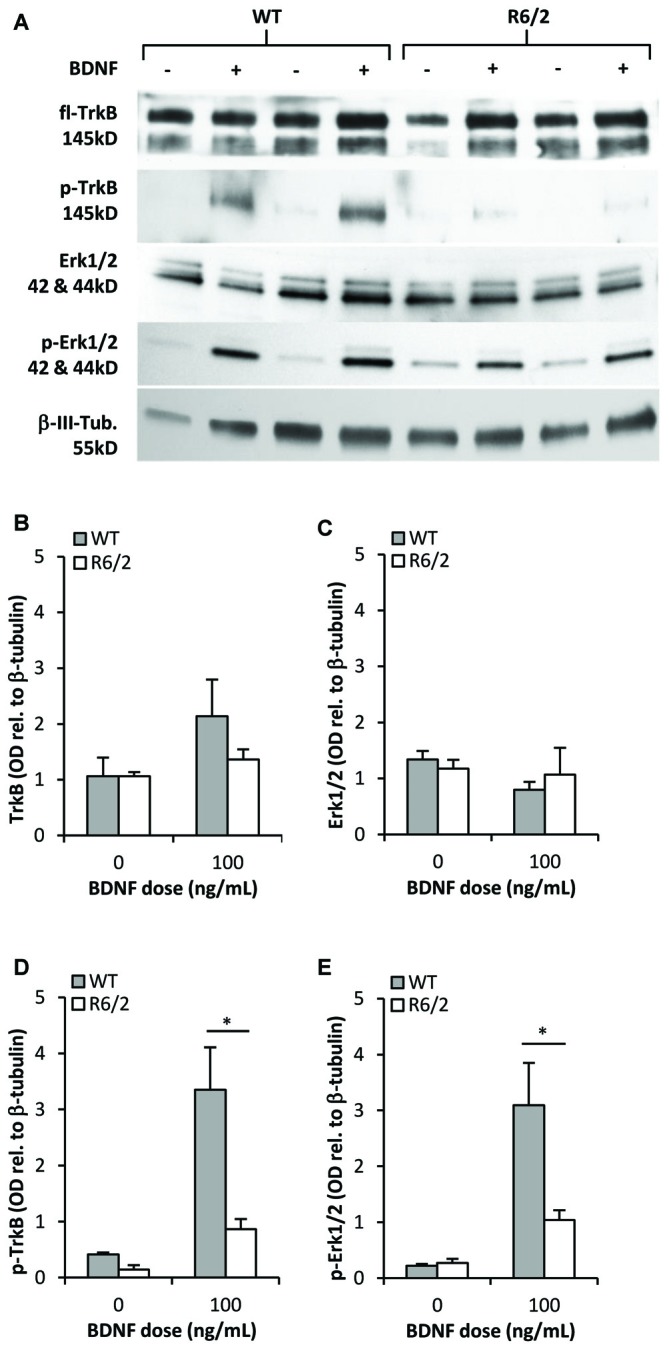
**TrkB activation and signal transduction is impaired in R6/2 striatal cultures. (A)** Sample immunoblots of TrkB and Erk1/2 signaling proteins from WT and R6/2 cultures after exposure to control (−) or 100 ng/mL BDNF (+) supplemented media. **(B)** fl-TrkB and **(C)** Erk1/2 levels are similar in control treated WT (*n* = 6) and R6/2 (*n* = 5) cultures. BDNF treatment did not change the levels of these proteins. **(D)** p-TrkB and **(E)** p-Erk1/2 levels after treatment with saline or BDNF. BDNF treatment increased p-TrkB and p-Erk1/2 levels to a greater extent in WT (*n* = 6) compared to R6/2 (*n* = 5) cultures. Relative protein levels were determined by normalization to the neuronal specific cytoskeleton protein β-tubulin. Unpaired Student’s *t*-test *p < 0.05.

We determined whether activation of the BDNF-TrkB signaling cascade is impaired in primary striatal cultures from R6/2 mice. Cultures grown for 48 h in N2-basal media were acutely (20 min) exposed to control or BDNF-supplemented media- a high dose of 100 ng/mL BDNF was used to maximize the detection of activated phospho-TrkB. In control conditions (saline) fl-TrkB (Figures 4A,B) and phospho-TrkB (Figures 4A,D) levels were similar in WT and R6/2 cultures. Exposure to BDNF elevated phospho-TrkB levels over control conditions in both WT and R6/2 cultures (Figures 4A,D). Importantly, BDNF yielded higher phospho-TrkB levels in WT compared to R6/2 cultures, indicating that BDNF-induced activation of TrkB is blunted in R6/2 cultures.

Since previous studies in cellular and animal models of HD have reported defects in BDNF-mediated activation of TrkB-Erk1/2 rather than the-Akt pathway (Ginés et al., 2010; Brito et al., 2013), we assessed the levels of activated phospho-Erk1/2 to characterize transduction downstream of TrkB in R6/2 cultures. In control conditions total Erk1/2 and phospho-Erk1/2 levels were similar in WT and R6/2 cultures (Figures 4A,C). Acute exposure to BDNF increased phospho-Erk1/2 levels over control conditions in both WT and R6/2 cultures (Figures 4A,E). However, BDNF yielded higher phospho-Erk1/2 levels in WT compared to R6/2 cultures, indicating that TrkB signal transduction down the Erk1/2 pathway is attenuated in R6/2 cultures.

## Discussion

The primary neuropathological feature of Huntington disease (HD) is an early and prominent degeneration of MSNs, the principal projection neurons of the striatum. Various gain- and loss-of-function mechanisms are proposed to underlie MSN degeneration in HD, including excitotoxicity, metabolic failure, altered protein expression and impaired signal transduction (Harjes and Wanker, 2003; Roze et al., 2011). Gain-of-function effects of *mhtt* are effectively assessed in the R6/2 mouse model of HD, which expresses an N-terminal fragment of *mhtt* in the presence of normal Htt protein and exhibits a phenotype of early striatal MSN degeneration and locomotor dysfunction (Mangiarini et al., 1996; Stack et al., 2007; Samadi et al., 2013). Studies in cellular and animal models of HD suggest various defects in the BDNF-TrkB signaling pathway may lead to reduced neurotrophic support of MSNs, but the precise nature and timing of these can vary depending on the model. The present study assessed striatal tissue and primary cultures from young R6/2 mice to determine whether early defects in the BDNF-TrkB pathway underlies reduced survival and maintenance of striatal MSNs. The main findings show striatal levels of phospho-TrkB are low in young R6/2 mice prior to any significant neuronal degeneration or loss of BDNF in the striatum. Additionally, *in vitro* experiments on R6/2 striatal cultures show that BDNF activation of phospho-TrkB and the Erk1/2 pathway is impaired in association with a reduction in BDNF-mediated neurotrophic effects on MSNs.

Studies on human HD tissue samples and YAC72, BACHD and R6/2 mice (Zuccato et al., 2001, 2005, 2008; Gray et al., 2008) show that cortical BDNF mRNA expression is impaired, suggesting this reduces BDNF supply to the striatum in these HD models. Conflicting studies report that cortical BDNF expression is normal in symptomatic BACHD mice (Plotkin et al., 2014) and human HD tissue samples (Ferrer et al., 2000; Gauthier et al., 2004), arguing that other downstream defects contribute to reduced BDNF neurotrophic support, such as impaired axonal transport machinery (Gauthier et al., 2004) or altered TrkB receptor signal transduction (Plotkin et al., 2014). Evidence of normal striatal BDNF levels at late disease stages in YAC72, R6/2 and other mouse models (Ginés et al., 2006; Pang et al., 2006; Seo et al., 2008; Cepeda et al., 2010; Bobrowska et al., 2011) further argues that impaired afferent BDNF supply alone may not account for impaired neurotrophic support. The current study extends evidence of normal striatal BDNF levels in young adult R6/2 mice, suggesting. downstream defects in TrkB receptor transduction may underlie early deficits in neurotrophic support of MSNs in this HD model.

Full-length TrkB receptors, whose expression is increased in the striatum during the perinatal period (Fryer et al., 1996), mediate BDNF’s neurotrophic effects on MSN survival and phenotypic maintenance in the developing and adult striatum (Alcántara et al., 1997; Ivkovic et al., 1997; Baydyuk et al., 2011). For example, germline or regionally targeted knockout of striatal fl-TrkB (Baydyuk et al., 2011; Li et al., 2012) attenuates the developmental rise of striatal DARPP-32 expression that occurs during the first postnatal month in rodents (Ehrlich et al., 1990). Moreover, ongoing TrkB-mediated regulation of striatal DARPP-32 expression is demonstrated in post-weanling mice (P24–31) that are exposed to cocaine (Niculescu et al., 2008). The R6/2 *mhtt* transgene is driven by a promotor analogous to the endogenous Htt gene, and the expression of Htt in CNS neurons increases during the first few postnatal weeks (Bhide et al., 1996). We assessed components of the TrkB pathway in adult R6/2 mice and primary cultures derived from postnatal striatum. TrkB expression is reduced in knock-in *mhtt* STHdhQ111 immortalized cells (Ginés et al., 2010). Furthermore, reduced levels of total TrkB are demonstrated in the striatum of young knock-in HdhQ111 mice (Ginés et al., 2006; Brito et al., 2013). This model retains a normal lifespan and exhibits only a subtle HD-phenotype of striatal degeneration. In contrast, several other *mhtt* knock-in and transgenic mouse models that exhibit clear striatal degeneration, show normal striatal total TrkB levels at both early and late disease stages (Brown et al., 2008; Gharami et al., 2008; Martire et al., 2010; Xie et al., 2010; Simmons et al., 2013; Plotkin et al., 2014; Smith et al., 2014; Ma et al., 2015). The present findings in young R6/2 mice show that striatal total TrkB levels are normal at a stage prior to striatal atrophy and neuronal loss. We now provide evidence that striatal levels of activated phospho-TrkB are reduced at this early stage when BDNF levels are normal. Striatal DARPP-32 levels are also reduced at this early stage in R6/2 mice. Indeed DARPP-32 expression is regulated by BDNF-TrkB signaling independent of neuronal survival (Ivkovic and Ehrlich, 1999). These findings suggest impaired activation of phospho-TrkB signaling is associated with reduced neurotrophic maintenance of MSN-phenotype at an early age in R6/2 mice.

Reduced BDNF-TrkB neurotrophic support of MSNs is further demonstrated in our experiments on R6/2 primary striatal cultures. In keeping with previous studies using primary striatal cultures derived from HD mice (Petersén et al., 2001; Liot et al., 2013), the basal neuron survival of R6/2 cultures was similar to WT cultures when grown in unsupplemented media. We now show that whereas BDNF-supplemented media promotes neuronal survival by up to ~34% in WT cultures, this effect is attenuated in R6/2 cultures to only ~21%. These *in vitro* findings suggest that MSNs in R6/2 mice are relatively resistant to pro-survival effects of BDNF. Additionally, while BDNF increases the population of DARPP-32 expressing cells by up to ~76% in WT cultures, this effect is attenuated in R6/2 cultures to only ~41%. Notably the BDNF effect on neuronal survival does not completely account for its greater effect on DARPP-32 expression, which is in line with studies suggesting parallel roles of BDNF in promoting survival (Nakao et al., 1995; Baydyuk et al., 2011) and maintaining phenotypic properties of MSNs (Ivkovic and Ehrlich, 1999). These *in vitro* findings indicate that BDNF maintenance of MSN phenotype is impaired in R6/2 mice. This is in keeping with our *in vivo* results showing an early decline of striatal DARPP-32 expression in young R6/2 mice, even in the presence of normal BDNF levels and total neuronal population.

BDNF-TrkB signal transduction involves several pathways, including Erk1/2, phosphoinositol-3 kinase (PI3K) and phospolipase C-gamma (PLCg; Reichardt, 2006). TrkB-mediated neuronal survival and maintenance of a wide variety of CNS neurons is dependent on the Erk1/2 pathway (Atwal et al., 2000; Cheng et al., 2002; Barnabe-Heider and Miller, 2003), and is a major pathway that is necessary for BDNF-dependent transcription and phenotypic maintenance of striatal neurons (Gokce et al., 2009). Studies in cellular models indicate that *mhtt* expression can elevate activation of Erk1/2 signaling (Apostol et al., 2006), which may be part of a pro-survival response or associated with dysfunction of other signaling pathways (Ribeiro et al., 2010; Bodai and Marsh, 2012). Significant variability is noted in different studies examining striatal Erk activation in transgenic or knock-in models of HD. Indeed some studies using R6/2 mice demonstrate normal striatal phospho-Erk1/2 levels at 8 weeks, and elevated levels are noted only at late stages (Liévens et al., 2002; Roze et al., 2008), possibly related to stress responses or dysfunctional transcriptional feedback (Liévens et al., 2002; Roze et al., 2008; Bodai and Marsh, 2012). In contrast other studies in R6/2 mice report reduced striatal phospho-Erk1/2 levels at 8 and 12 weeks (Fusco et al., 2012; Simmons et al., 2013). Importantly, TrkB-mediated activation of the Erk1/2 pathway is impaired in other cellular and mouse models of HD. For example *mhtt* knock-in STHdhQ111 cell lines or HdhQ111 mouse-derived primary cultures exhibit attenuated BDNF-mediated activation of phospho-Erk1/2 (Ginés et al., 2010; Liot et al., 2013). It was suggested that impaired expression or transport of TrkB may account for attenuated BDNF-mediated Erk1/2 signaling in these models. The final level of Erk1/2 activation is likely dependent on multiple factors altered in HD striatum (e.g., dopamine, glutamate signaling; Gardoni and Bellone, 2015), which act in concert with alterations related to the BDNF-TrkB survival pathway. We now provide evidence that basal levels of TrkB, phospho-TrkB, Erk1/2 and phospho-Erk1/2 are normal in R6/2 striatal cultures. However, BDNF activation of phospho-TrkB and phospho-Erk1/2 is attenuated, and is associated with reduced BDNF-mediated survival and phenotypic maintenance of R6/2 cultures. These results are consistent with the observed deficiencies in striatal TrkB activation and phenotypic maintenance in the presence of normal BDNF levels in early presymptomatic R6/2 mice.

The cause of striatal degeneration in HD is likely related to multiple cell autonomous and non-autonomous mechanisms, including excitotoxicity in conjunction with impaired neurotrophic support (Harjes and Wanker, 2003; Milnerwood and Raymond, 2010; Baydyuk and Xu, 2014). Since the striatum does not produce BDNF and depends on supply from cortical, thalamic and midbrain afferents (Altar et al., 1997; Sadikot et al., 2005), anterograde neurotrophic factor support may be critical for striatal neurons in HD (Zuccato et al., 2005). The present study demonstrates that striatal neurons from the R6/2 model of HD also exhibit a blunted trophic response to BDNF that is associated with decreased activation of the TrkB-Erk1/2 signaling pathway. This work suggests neurotrophin-based therapies for HD should address both the deficit in BDNF supply (Bates et al., 2015) and the impaired signal transduction from the TrkB receptor (Apostol et al., 2008; Simmons et al., 2013). Further characterization of *mhtt* interactions with TrkB signaling pathways may contribute to development of novel targets for therapy in HD.

## Author Contributions

All authors listed, have made substantial, direct and intellectual contribution to the work, and approved it for publication.

## Conflict of Interest Statement

The authors declare that the research was conducted in the absence of any commercial or financial relationships that could be construed as a potential conflict of interest.

## Abbreviations

BDNF: brain-derived neurotrophic factor
DARPP-32: dopamine- and cAMP-regulated neuronal phosphoprotein 32 kDa
Erk1/2: extracellular signal-regulated kinase 1/2
htt: huntingtin
HD: Huntington disease
MSNs: medium spiny neurons.
